# Asymmetry Upper Eyelid Retraction of Thyroid Eye Disease: Change of Lateral Flare Sign With the Natural Course of Untreated Disease

**DOI:** 10.1155/sci5/1855515

**Published:** 2025-05-23

**Authors:** Shiqi Hui, Ju Zhang, Zhijia Hou, Dong-Mei Li

**Affiliations:** ^1^Beijing Tongren Eye Center, Beijing Tongren Hospital, Capital Medical University, Beijing, China; ^2^Aier Eye Hospital Group Co., Ltd. Beijing Aier Eye Hospital, Jinan University, Guangzhou, Guangdong, China

**Keywords:** asymmetrical upper eyelid retraction, multiple radial midpupil lid distance, natural course of disease, photographic analysis, thyroid eye disease

## Abstract

**Purpose:** To assess the correlation of upper eyelid retraction and the disease natural course of untreated thyroid eye disease patients, using multiple radial midpupil lid distance, for individualized eyelid morphological treatment.

**Methods:** Semiautomatic photographic analysis was performed using ImageJ. Temporal/nasal multiple radial midpupil lid distance ratios of the same angles with respect to the midline was calculated. The influence of several variable factors on the asymmetrical upper eyelid retraction was evaluated.

**Results:** One hundred and fifty-one eyes of 85 untreated thyroid eye disease patients were included. The mean age of patients was 45.4 ± 14.7 years. Multiple radial midpupil lid distance ratios increased during the first 12 months, 105°/75° (*r* = 0.196, *p*=0.087), 120°/60° (*r* = 0.250, *p*=0.028), 135°/45° (*r* = 0.309, *p*=0.006), and 150°/30° (*r* = 0.275, *p*=0.015). The ratios then slowly decreased, 120°/60° (*r* = −0.332, *p*=0.006), 135°/45° (*r* = −0.297, *p*=0.014), and 150°/30° (*r* = −0.254, *p*=0.037). The ratios plateaued at 13 months but were still greater than normal range. No correlation found between smoking history (*p*=0.230), family history (*p*=0.382), exophthalmometry (*p*=0.597), and natural course. Women had a higher likelihood of upper eyelid retraction asymmetry, 120°/60° (*p*=0.041) and 135°/45° (*p*=0.048). The absence of systemic disease was associated with a lower likelihood of upper eyelid retraction asymmetry in 120°/60° (*p*=0.036). No significant difference was found in the contralateral eye of unilateral patients.

**Conclusion:** Multiple radial midpupil lid distance is a valuable method for measuring asymmetrical upper eyelid retraction, enabling improved understanding in upper eyelid contour with the duration of untreated thyroid eye disease. Allowing a better decision on individualized treatment.

## 1. Introduction

Thyroid eye disease (TED) being a multifactorial autoimmune disease involves orbital and periorbital tissues' inflammation with clinical signs and symptoms [1]. Surgery, oral and intravenous glucocorticoid therapy, nonsteroidal oral immunosuppressive agents, and immunomodulatory monoclonal antibodies are the current mainstream treatment options. The management of TED remains complex, considering a multitude of factors including patient preference, age, gender, pregnancy, and smoking.

Eyelid retraction is a prime diagnostic criterion for TED. Upper eyelids resting at or above the limbus is considered as retracted [2]. Upper eyelid retraction occurs in as many as 90% patients, and it often shows contour abnormalities [3, 4]. Lateral lid flare is a well-known contour deformity of TED [5], a key element of the surgical technique. Treatment of eyelid retraction in TED patients is still a challenging and controversial aspect of ophthalmic plastic surgery. Upper eyelid retraction is associated with exposure to keratopathy and may result in sight-threatening, in addition to its esthetic alterations, which may lead to further psychosocial implications and affect patient's quality of life. Moreover, under- or overcorrection of eyelid may lead to adverse outcomes and increased medical burden. Thus, a better understanding of the tomography of upper eyelid retraction in TED patients is crucial for further management and individualized treatment.

Objective assessment of eyelid measurement is essential for disease diagnosis, medical recording, observation of therapeutic effects, and communication in multicenter research interventions. Studies have proved semiautomatic measurement is a highly repeatable and reproducible scheme for topographic analysis of the eyelid position [6–9]. Multiple radial midpupil lid distance (MPLD), first described by Milbratz et al. [10] can quantify and graphically display contour abnormalities, provides a more accurate description on outlining the lid contour than the commonly used margin reflex Distance 1, which is the vertical distance between midpupil to lid margin.

Most TED individuals with untreated upper eyelid retraction have spontaneous improvement [11]. But few studies described the improvement of upper eyelid retraction with disease duration in TED patients. In this study, we measured MPLD ratio to describe the asymmetrical upper eyelid retraction contour in TED patients. We then observed the change of MPLD ratio with disease duration for a better option of choosing interfering timing on the asymmetrical eyelid retraction. The purpose of this study was to evaluate the correlation between MPLD ratio, associated clinical factors, and natural course of untreated TED for individualized treatment.

## 2. Materials and Methods

### 2.1. Participants and Study Design

This investigation was a retrospective, noninterventional study using photographs from 130 patients with TED distributed across China's mainland. Patients were aged 10–73 years and referred to the Department of Ophthalmology of Beijing Tongren Eye Center, Beijing Tongren Hospital, Capital Medical University from December 2020 to January 2023. The IRB/Ethics Committee has ruled that approval was not required for the study.

Patients taking medication, with history of eyelid surgery, orbital surgery, or history of myasthenia were excluded from the study. Patients diagnosed with strabismus were excluded. Digital photographs lacking in quality were excluded. Of 130 patients recruited, 85 fulfilled the inclusion criteria.

All patients had a digital photograph taken by the same person using SONY DSC-F828 camera (Sony Group Corp., Tokyo, Japan) positioned in the frontal plane at pupil height, 1 m from the patient. A dark blue background was used under artificial light. Patients were instructed to relax; remove makeup, jewelry, and glasses; and focus on the center of the camera lens with eyelid at the normal resting position. Hair was fixed at the back. Flash light was not used to avoid blinking of the eyes.

### 2.2. Image Processing

The digital photographs were transferred to a personal computer and saved as JPG files (3264 × 2448 pixels). Camera settings were as follows: aperture f/2.2, shutter speed, 1/40 s, ISO-64, and 9 mm focal length.

**Figure 1:**
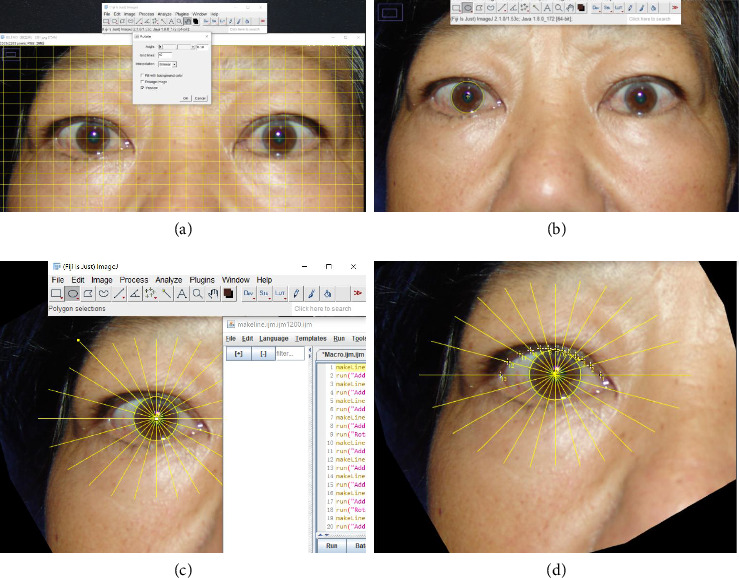
Lid contour analysis. (a) Digital photograph adjustment. (b) The center by using the ellipse fitting process. (c) Eleven oblique MPLDs. (d) Plot intersections.

Digital photographs were analyzed with ImageJ [12]. Photographs were adjusted to an exact horizontal orientation by reference to the canthal position (Figure 1(a)), then cropped automated for further processing. The center of cornea was located by using the ellipse fitting process (Figure 1(b)). We assumed that the cornea and pupil shared concentric circles.

We created a macro to automatically draw 11 oblique MPLDs on each 15° of the eyelid margins (Figure 1(c)). The nasal sector was at 75°, 60°, 45°, 30°, and 15°; the temporal sector was at 105°, 120°, 135°, 150°, and 165°; and the vertical line was at 90°. Because canthus location varies among individuals, the medial canthal was located between −15° and 15°, whereas the lateral canthal was between 165° and 195°. Therefore, the ratio of 0° and 180° were excluded from this study. We manually plotted intersections of the radial lines on the upper eyelid margin (Figure 1(d)). The radial MPLD lengths were measured, and the data were exported in csv format. The temporal/nasal MPLD ratios were calculated at the same angles with respect to the midline (105°/75°, 120°/60°, 135°/45°, 150°/30°, and 165°/15°).

Measurements of MPLD were performed by two investigators. If no significant difference from their measurements was observed, mean measurement was taken for further investigation. If a significant difference was found, investigators were asked for repeated measurements. The medical records of all patients were retrospectively reviewed, which included age, sex, smoking history, family history of thyroid disease, systemic disease, disease duration, clinical activity score (CAS), and exophthalmometry (Hertel Exophthalmometry). We assessed the correlation of change in upper eyelid retraction with disease duration, CAS, and exophthalmometry. Exophthalmometry was measured with computed tomography images using ImageJ.

The CAS (score 0–7) amended by the European Group on Graves' Orbitopathy was assessed by an experienced ophthalmologist to evaluate the clinical activity and degree of inflammation in each eye. The severity of CAS was estimated according to the initial classical signs and symptoms: pain, redness, and swelling of the eye and appendages. Orbit with CAS score of three or more were classified as active eye, whereas orbit with scores less than three were inactive eye. Duration of TED was defined as the time between each visit in our consult room and the first recall of eye symptoms.

### 2.3. Statistical Analysis

SPSS (SPSS 20.0 for Windows; SPSS Inc., Chicago, IL, USA) was used for statistical analysis. A one-way analysis of variance was used to evaluate the differences in the measurement data provided by two investigators. The comparison of CAS and exophthalmometry between before and after the turning point of the 12th month according to the natural course of untreated disease performed with the Mann–Whitney *U* test. The comparison of categorical data such as sex, laterality (left/right eyes), family history, systemic history, and smoking history before and after the turning point of 12th month according to the natural course of untreated disease was performed with Pearson's Chi-square test. Correlations between MPLD ratio, CAS, exophthalmometry, and disease duration were evaluated by Spearman's test. Comparisons of the same patients between two eyes were analyzed using the paired *t*-test. Correlations between influence factors of upper eyelid retraction asymmetry and MPLD ratio were analyzed using stepwise linear regression. All tests were two-tailed; a *p* < 0.05 was considered to indicate statistical significance.

## 3. Results

We included 151 eyes from 85 patients in this study. Seven of the 52 (13.5%) women and 23 of the 33 (69.7%) men had smoking history. The mean age was 45.4 ± 14.7 years. The mean disease duration was 26.6 ± 32.7 months.  Table 1 shows comparison before and after the turning point of 12th month according to the natural course of untreated diseases. There was a significant reduction in activity in TED (*p*=0.012). No significant difference was found between the measurements of our two investigators (*p*=0.916).

## 4. Natural Course of Upper Eyelid Retraction Contour Asymmetry

**Figure 2:**
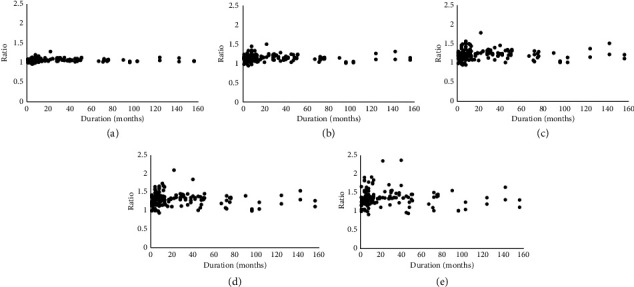
Correlation between natural course and MPLD ratios. (a) 105°/75°, (b) 120°/60°, (c) 135°/45°, (d) 150°/30°, and (e) 165°/15°.

**Figure 3:**
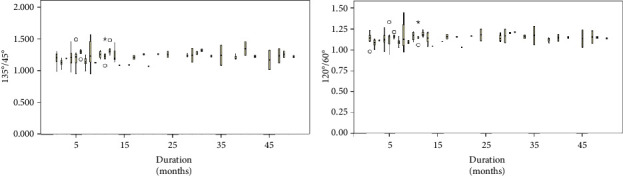
Box plot of data on 120°/60° and 135°/45° MPLD ratios with the disease natural course from 1 to 50 months.

Figure 2 shows the natural course of MPLD ratios for 156 months. The disease duration is plotted on the *x*-axis. The lid measurements and the asymmetry ratio were plotted on the *y*-axis. MPLD ratios were correlated positively with duration from 0 to 12 months, 105°/75° (*r* = 0.196, *p*=0.087), 120°/60° (*r* = 0.250, *p*=0.028), 135°/45° (*r* = 0.309, *p*=0.006), 150°/30° (*r* = 0.275, *p*=0.015), and 165°/15° (*r* = 0.186, *p*=0.105). The ratios reached a peak at 12 months, then slowly declined without reaching the normal range by 156 months, 105°/75° (*r* = −0.236, *p*=0.053), 120°/60° (*r* = −0.332, *p*=0.006), 135°/45° (*r* = −0.297, *p*=0.014), 150°/30° (*r* = −0.254, *p*=0.037), and 165°/15° (*r* = −0.149, *p*=0.225). Box plot of data on 120°/60° and 135°/45° MPLD ratios with the disease natural course from 1 to 50 months is shown in Figure 3.

### 4.1. Associated Factors

**Figure 4:**
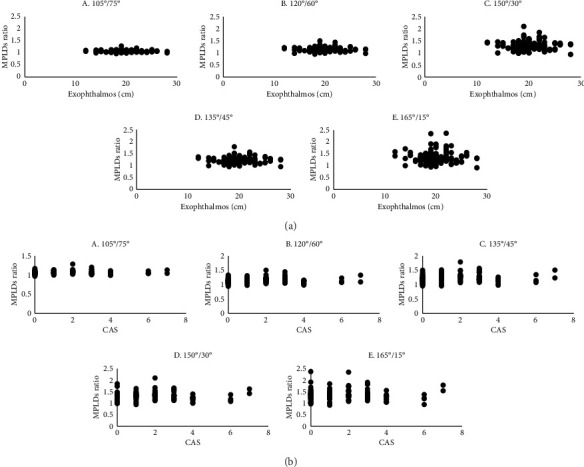
Correlation between MPLD ratios and exophthalmos (a); MPLD ratios and CAS (b).

A higher statistical correlation was observed in 120°/60° and 135°/45° MPLD ratios with the natural course of untreated diseases, we further analyzed the correlation between associated factors and MPLD ratios (Table 2). Women had a greater likelihood of having contour asymmetry compared with men at 120°/60° MPLD ratios (*p*=0.041; *R*^2^ = 0.107; D-W = 1.971; VIF < 2) and at 135°/45° MPLD ratios (*p*=0.048; *R*^2^ = 0.091; D-W = 1.912; VIF < 2). Lack of systemic disease was associated with a low likelihood of contour asymmetry at 120°/60° (*p*=0.036; *R*^2^ = 0.107; D-W = 1.971; VIF < 2). No significant association was observed between exophthalmos (Figure 4(a)), CAS (Figure 4(b)), and MPLD ratios.

### 4.2. Comparison Between Lateral Eyes

**Figure 5:**
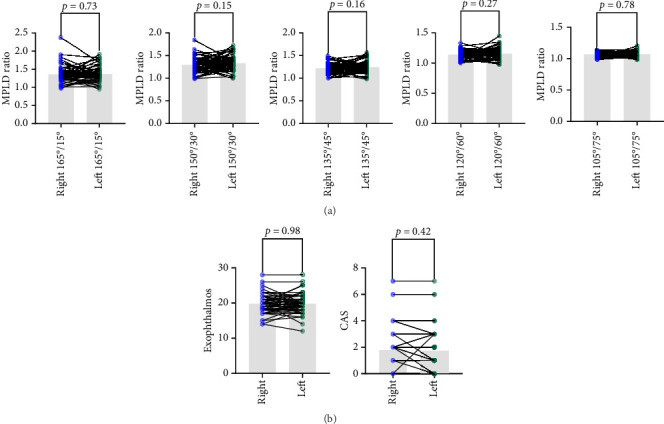
Comparison of MPLD ratios and exophthalmos between two eyes of the same patient.

We compared MPLD ratios (Figure 5(a)), CAS, and exophthalmometry (Figure 5(b)) of the contralateral eye of the same patient. No significant difference was found among them.

## 5. Discussion

In this study, we demonstrated the procession of a lateral flare sign with untreated natural course in TED patients. Clinical factors including but not limited to sex, exophthalmos, and systematic disease are found to be associated with the degree of the lateral flare sign through stepwise linear logistic. These results suggest that a predictive model of upper eyelid contour of TED patients can be established in the near future after bettering clinical records and expanding our dataset.

Upper eyelid retraction is the most common clinical sign of TED. The mechanism of retraction remains controversial [13, 14]. A better understanding of how upper eyelid retraction behaves during the course of untreated disease is important for clinician to counsel patients on further management. Self-limited processes of upper eyelid retraction in untreated TED are found in more than 50% of patients with TED, and the esthetic alterations caused by upper eyelid retraction can still result in decreased confidence and increased social isolation, which affects mood, increases anxiety and depression among patients [15, 16]. However, correction of eyelid retraction is difficult. Methods vary for correcting upper eyelid retraction, such as antithyroid drugs, radioactive iodine, thyroidectomy, and injection of botulinum toxin, hyaluronic acid filler, triamcinolone acetonide, and emerging biologic therapies [17–19]. Upper eyelid retraction repairment in unilateral patients may induce an elevation of the contralateral upper eyelid. But so far, there is no guarantee in avoiding after-treatment side effects and reoperation leading to adverse outcomes and increased costs. Therefore, it is necessary to have a better understanding on upper eyelid retraction spontaneous improvement in TED for planning individualized treatment may allow nonsurgical modalities valuable alternatives in specific cases, resulting in more successful outcomes.

There are limited reports that describe the course of upper eyelid retraction in TED. Lee et al. [20] suggested that more than 50% eyes with upper eyelid retraction in patients with TED improved by 6 months. Univariable logistic regression showed that the position of the upper eyelid was significantly associated with exophthalmos, CAS score, and thyroid hormone immunoglobulin. Other investigators have described mainly on ocular changes other than upper eyelid retraction in patients with TED [21, 22].

In our study, the lateral flare sign correlated positively with duration from 0 to 12 months and reached a peak at 12 months, then slowly declined without reaching the normal range by 156 months. Women had a significantly higher likelihood of having contour asymmetry compared with men. Hormone levels may play a role in the sexual difference, affecting eye tissue edema and inflammatory response, leading to different clinical manifestations [23]. According to previous findings, women are often more sensitive to immune reactions than men [24]. Lack of systemic disease was observed to be associated with a lower likelihood of having a lateral flare sign. There was no significant difference found in the natural course of untreated upper eyelid retraction among eyes with and without eyelid inflammation, and the spontaneous improvement occurred regardless of disease activity. In terms of factors associated with the occurrence of upper eyelid retraction asymmetry, a higher attention is suggested to pay on counseling these patients. Furthermore, we did not observe an association of MPLD ratios with exophthalmos. This lack of association may be explained by only extreme degrees of proptosis exacerbate the retraction mechanically [23].

TED is a bilateral disease, but eyes may be affected unilaterally. In our study, 21.18% patients (18 out of 85) had the asymmetry effect in between the two eyes. This may be explained by unilateral disease only account for 9%–15% from previous studies [24]. Among these patients, no significant difference was found in CAS score, lid flare sign, or exophthalmometry between their two eyes. Based on the available literature, euthyroid patients with TED was more likely to present as unilateral ophthalmopathy [25]. Thus, thyroid function may have a higher weight on unilateral patients. The course of untreated disease in unilateral patients in this study varies from 3 to 90 months, and we tend to look into their fellow eye in follow-up to see whether they are affected over time in the further study.

A linear measurement such as margin reflex distance or MPLD could not represent the exact amount of the retracted eyelid over the curved surface of the eye. MPLD ratios can clearly describe the lateral flare sign while minimizing the small difference between curved and linear measurements, without assuming arbitrary corneal diameters reference to pixel widths. We used calculated center for measurement instead of the light reflex on the cornea, for the cornea's optical reflection point is not adequate for clinical work. The point can be affected by unaligned lighting, strabismus, and other factors.

Our study had limitations. In the assessment of the eyelid position, a single photograph of the eyelid may not be as accurate as an average value for the eyelid position based on multiple video picture frames. Canthus to canthus distance was not considered in the current study, as it may affect the measurement results. In addition, the patients we evaluated were treated at a single hospital, and though we absorb patients from all over the country, there might be selection bias. Untreated TED patients with a duration over 50 months are not commonly seen, thus leading to a limited sample size, which may lead bias to our result. Though semiautomated, ImageJ is highly reproducible for the display of contour abnormality. Artificial intelligence–assisted image processing algorithm for automated eyelid measurement was brought by Chen et al. [26]. Although the performance of artificial intelligence in data analysis is promising, the real performance still remains unclear because of the difficulty of interference factors such as eyelashes and nonuniform luminance background (white sclera, black pupil, and a gradient of iris colors) have on finding the eyelid margin.

Due to its potential for facial disfigurement and negative impact on quality of life, TED remains a challenging condition. An additional study of the natural course of upper eyelid retraction in untreated TED should measure contour change during long-term follow-up. Our findings provide a valuable method to measure the contour asymmetry of upper eyelid retraction and an improved understanding of the change in upper eyelid topography with disease duration.

## 6. Conclusion

Multiple radial MPLD is a valuable method in measuring the tomography asymmetry of upper eyelid retraction. This method enables improvement in understanding the change of upper eyelid contour with disease duration in TED patients, as well as the further management of disease. The peak amplitude of the lateral lid flare sign was observed at 120°/60° and 135°/45° of MPLD ratios. Improved understanding in the lateral lid flare sign with the duration of untreated TED allowing a better decision on individualized treatment.

## Figures and Tables

**Table 1:** Comparison before and after the turning point of the 12th month according to the natural course of untreated disease.

Characteristic	Before 12 months	After 12 months	*p*
Number of eyes (*N*)	77	74	
Sex (male: female)	17:25	16:27	0.826^†^
Age (years)	46.5 (31–57.25)	47 (36–57)	0.622^∗^
Laterality (left: right)	40:37	37:37	0.871^†^
Exophthalmometry (cm)	19.5 (18–21)	19 (18–22)	0.597^∗^
Smoking status (current smoker: Nonsmoker: Ex-smoker: Passive smoker)	21: 40: 8: 8	12: 38: 10: 14	0.230^†^
Clinical activity score (CAS)	2 (0–3)	1 (0–2)	0.091^∗^
Activity (CAS < 3: CAS ≥ 3)	48:29	60:14	0.012^†^
Family history (yes: no)	8:34	5:38	0.382^†^
Systemic disease (yes: no)	14:28	9:34	0.229^†^
Natural course (months)	6 (4–8)	36.5 (20–72)	

*Note:* Values are expressed as medians with an interquartile range for measurement data and ratio for enumeration data. The bold value indicates significance at *p* < 0.05.

^∗^Mann–Whitney *U* test.

^†^Pearson's Chi-square test.

**Table 2:** Influencing factors on upper eyelid retraction asymmetry in 120°/60°(A) and 135°/45°(B).

(A) (*p*=0.047^∗^) (95% Cl)		Nonstandardization coefficient		Standardization coefficient	*t*	*p*
		B	SE			
(Constant)		1.033	0.078		14.582	< 0.001

Sex	Male	0	0.021	0.185	1.605	**0.041** ^**∗**^
	Female	0.033				

Proptosis		0.002	0.003	0.066	0.724	0.470

CAS		0.003	0.005	0.055	0.622	0.530

Systemic disease	Yes	0	0.019	−0.178	−1.857	**0.036** ^**∗**^
	No	−0.034				

Age (years)		0.001	0.001	0.090	0.919	0.360

Smoking status	Current	−0.023	0.021	−0.113	−1.090	0.278
	Ex	0.001	0.013	0.011	0.105	0.916
	Passive	0.008	0.008	0.095	1.023	0.308
	Non	0				

**(B) (** **p**=0.050^∗^**)** **(95% Cl)**		**Nonstandardization Coefficient**		**Standardization Coefficient**	***t***	**p**
		**B**	**SE**			

(Constant)		1.055	0.122		8.654	< 0.001

Sex	Male	0	0.032	0.184	1.582	**0.048** ^**∗**^
	Female	0.051				

Proptosis		0.004	0.004	0.088	0.959	0.339

CAS		0.003	0.008	0.037	0.418	0.676

Systemic disease	Yes	0	0.029	−0.144	−1.180	0.120
	No	−0.034				

Age (years)		< 0.001	0.001	0.029	0.297	0.767

Smoking status	Current	−0.031	0.033	−0.096	−0.920	0.359
	Ex	0.005	0.021	0.025	0.234	0.815
	Passive	0.012	0.012	0.099	1.048	0.297
	Non	0				

*Note:* The bold^∗^ values indicate significance at *p* < 0.05 using stepwise linear regression and stepwise linear figure.

Abbreviation: SE, standard error.

## Data Availability

The datasets used and analyzed during the current study are available from the corresponding author on reasonable request.
